# Some Like It Hot –Structural Changes in Extremophile Rubredoxin at 120 °C

**DOI:** 10.1002/anie.202520302

**Published:** 2025-11-24

**Authors:** Tzanko Doukov, Igor Leontyev, Francis E. Jenney, Dominic George, Stephen P. Cramer

**Affiliations:** ^1^ SSRL SLAC National Laboratory Menlo Park CA 94025 USA; ^2^ SETI Institute Mountain View CA 94043 USA; ^3^ Georgia Campus, Philadelphia College of Osteopathic Medicine Suwanee GA 30024 USA

**Keywords:** Extremophile, Molecular dynamics, *Pyrococcus furiosus*, Rubredoxin, X‐ray diffraction

## Abstract

How does the structure of a protein change as the temperature is raised from cryogenic conditions at 100 K to 393 K? Understanding the structure and dynamics of proteins under environmental extremes is relevant for human health, biotechnological applications, and our search for life elsewhere in the universe. Here we reveal the high temperature crystal structure of a hyperthermophilic (*Pyrococcus furiosus*) rubredoxin at 393 K (120 °C), together with multiple complementary structures down to 100 K. The results are compared with molecular dynamics calculations. Significant changes in H‐bonding are observed. Discussions about high‐temperature protein structure and stability need to recognize that low temperature structures may not represent the high temperature case.

## Graphical abstract

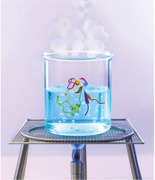

## Introduction

For life to thrive under extreme conditions,^[^ ^1^, ^2^ ^]^ its constituent proteins need to maintain stable and biochemically active structures.^[^ ^3^ ^]^ Understanding the structure of extremophile proteins under such conditions is interesting in its own right, and it is also relevant for human health, biotechnology, and our search for life elsewhere in the universe.^[^ ^4^, ^5^, ^6^, ^7^, ^8^ ^]^

A sterling example of high‐temperature protein stability is the rubredoxin (Rd)^[^ ^9^ ^]^ from the hyperthermophile *Pyrococcus furiosus* (*Pf*), a marine archaeon with an optimal growth temperature of ∼100 °C.^[^ ^10^ ^]^ *Pf* Rd is an electron donor to a superoxide reductase (SOR),^[^ ^11^ ^]^ using electrons supplied by NADPH via NADPH: rubredoxin oxidoreductase.^[^ ^12^ ^]^ *Pf* Rd is reported to have equilibrium melting transition *T*_m_ just below 200 °C,^[^ ^13^ ^]^ or at least *T*_m _= 144 °C^[^ ^14^ ^]^ For decades it has been intensely studied in an effort to identify the sources of its exceptional stability,^[^ ^15^, ^16^, ^17^, ^18^, ^19^, ^20^, ^21^ ^]^ which has been attributed to (a) the cluster of 6 aromatic residues at the *Pf* Rd core, (b) the metal‐binding site, (c) the H‐bonding in the three β‐sheets, and (d) the Glu‐15 bonding network. However, Petsko has warned, “*it is somehow unsatisfying to conclude that a single great property like extreme thermostability arises from a combination of many different small contributions*”.^[^ ^22^ ^]^

The Fe(S‐Cys)_4_ metal‐binding site of rubredoxins can have the native Fe replaced by numerous divalent metals, including Co, Ni, Ga, Cd, Hg,^[^ ^23^ ^]^ Cu,^[^ ^24^ ^]^ and Zn.^[^ ^25^ ^]^ To shed additional light on the puzzle of *Pf* Rd stability, here we report an x‐ray diffraction (XRD) structure at 393 K (120 °C) of Zn‐substituted *Pf* Rd, referred to hereafter as Zn *Pf* Rd. This temperature is 30 °C above the previous highest temperature structure in the PDB.^[^ ^26^ ^]^ An XRD structure has been reported for Fe *Pf* Rd at ∼100 K, ^[^ ^27^ ^]^ along with a neutron diffraction structure at room temperature;^[^ ^20^ ^]^ these are complemented by room temperature NMR structures of Zn *Pf* Rd.^[^ ^28^ ^]^ For comparison, we present 15 additional XRD structures at temperatures from 100 K to 383 K. This allowed us to examine the temperature dependent evolution of B‐factors, observable bound waters, and protein volume. Finally, the diffraction structures are compared with molecular dynamics (MD) calculations on the 5 µs time scale.

## Results

**Figure 1:**
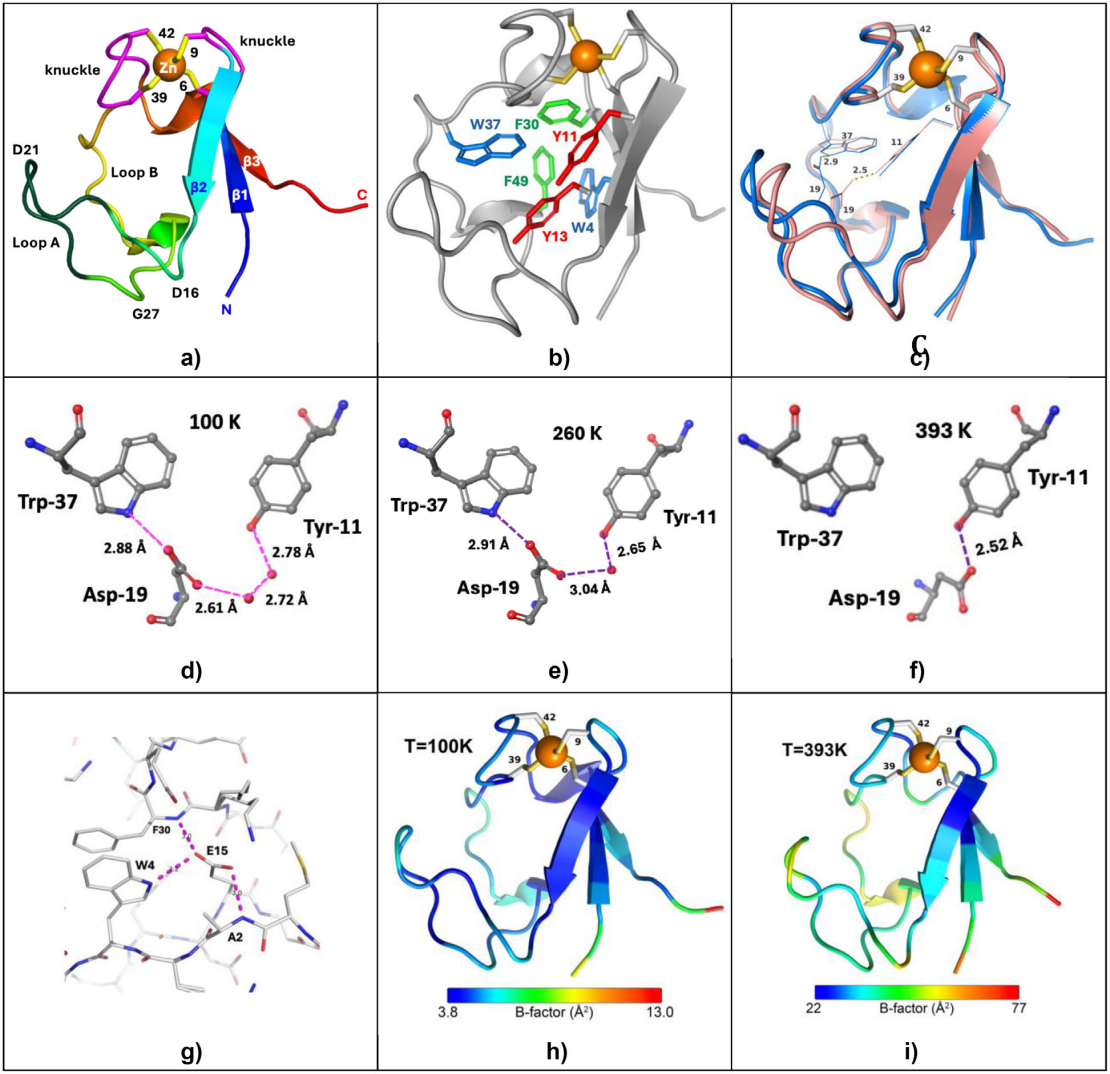
a) Ribbon diagram for *Pf* Rd highlighting key features. The darker green region is sometimes called the hairpin turn. b) Pymol cartoon accentuating aromatic amino acids in Zn *Pf* Rd at 100 K. c) Overlap of 100 K (blue) and 393 K (rose) x‐ray structures highlighting changes in H‐bonding for D19. d) Closeup of H‐bonding near D19 at 100 K. e) H‐bonding near D19 at 260 K. f) H‐bonding near D19 at 393 K. g) H‐bonding near E15 at 393 K. h) 100 K structure colored according to B‐factors. i) 393 K structure colored according to B‐factors.

Rubredoxins from psychrophiles, mesophiles, and hyperthermophiles share many common features, including the sequence similarities that are summarized in Table 1, and the general structure illustrated in Figure 1a. Along with metal‐binding “knuckles”,^[^ ^13^ ^]^ common structural features include a three‐stranded antiparallel β‐sheet and a stretch of amino acids that forms a loop from Asp‐16 to Gly‐27. A second loop runs from Phe‐30 to Val‐38 and we refer to these respectively as loop A and loop B. A key feature of Rds is a central hydrophobic core of 6 aromatic (or hydrophobic) residues shown in Figure 1b. In the *Pf* Rd structure with a terminal methionine, these amino acids are Trp 4, Tyr 11, Tyr 13, Phe 30, Trp 37, and Phe 49. For clarity and ease of comparison, we employ this same numbering for the *Pf* Rd with N‐terminal formyl‐methionine.

**Table 1:** Sequence comparisons for psychrophile (*Polaromonas glacialis – Pg*), mesophile (*Clostridium pasteurianum – Cp*), and hyperthermophile (*Pyrococcus furiosus – Pf*). Rds from organisms that respectively grow near 0 °C, 50 °C, and 100 °C. Conserved cysteines highlighted C, conserved prolines P, core aromatics highlighted W, Y, and F.

*Pg*	MT**W**M**C**LI**C**G**W**I**Y**DEALGS**P**EHGIAAGTP**W**SQV**P**MN**W**T**CP**E**C**GARKED**F**EMVQM
*Cp*	MKK**Y**T**C**TV**C**G**Y**I**Y**NPEDGD**P**DNGVNPGTD**F**KDI**P**DD**W**V**CP**L**C**GVGKDQ**F**EEVEE
*Pf*	MAK**W**V**C**KI**C**G**Y**I**Y**DEDAGD**P**DNGISPGTK**F**EEL**P**DD**W**V**CP**I**C**GAPKSE**F**EKLED

### The Zn Binding Site

The metal‐binding site of rubredoxin involves 4 cysteine thiolate ligands in a tetrahedral geometry. The site has been described as a C_2_‐pseudo‐symmetric fold made by two symmetry‐related CXXCX α‐turns – the so‐called “knuckles”.^[^ ^13^ ^]^ We employed Zn *Pf* Rd protein for the current study, to avoid ambiguities from possible X‐ray radiolysis of Fe *Pf* Rd under high temperature conditions. To quantify the overall similarity between the Fe(II) *Pf* Rd and Zn *Pf* Rd, we superimposed the two 100 K structures using all main chain atoms. The resulting RMSD for these atoms was 0.13 Å and the metals came within 0.04 Å of each other. The average Zn–S distance was 2.34 Å for Zn *Pf* Rd with an Ala terminus and 2.35 Å for the f‐Met version compared to the reported average Fe(II)‐S distance of 2.32 Å.^[^ ^29^ ^]^ Overall, the Zn *Pf* Rd should be a good proxy for Fe(II) *Pf* Rd.

### Temperature‐Dependent Crystal Structures of *Pyrococcus Furiosus* Rubredoxin

In Figure 1c we compare the structures obtained for the Zn *Pf* Rd between 100 K and 393 K. We see that the essential structure is maintained. However, there is one significant difference in the position of loop A, presumably due to a change in H‐bonding for Asp‐19. At low temperatures Asp‐19 is H‐bonded to Trp‐37, while at the highest temperatures it shifts to H‐bonding with Tyr‐11. These changes are illustrated in more detail in Figure 1d–f. At 100 K, one carboxylate O from Asp‐19 is within 2.88 Å of the Trp‐37 indole N, but it is also connected through the other carboxylate O to Tyr‐11 by a pair of water molecules. At 260 K, there is a similar connection to Trp‐37, but only a single water link to Tyr‐11. Finally, at 393 K, the H‐bonding to Trp‐37 is lost and the Asp‐19 carboxylate has rotated to make a direct H‐bond with Tyr‐11.

Another set of H‐bonding interactions occurs near the N‐terminus of *Pf* Rd. Based on NMR, Blake proposed in 1991 “potentially stabilizing electrostatic interactions involving the charged groups of residues” Ala‐2, Glu‐15, and Glu‐53 (our numbering).^[^ ^30^ ^]^ Jung and others^[^ ^31^ ^]^ have noticed that the flexibility of loop A is reduced by “multiple electrostatic interactions of Glu‐15”^[^ ^31^ ^]^ and claim the hydrogen bond between Glu‐15 and Ala‐2 remains even at high temperature. Neutron diffraction studies have also characterized electrostatic interactions around the N‐terminal that are proposed to contribute to its stability, including the hydrogen bond between Glu‐15 and Ala‐2.^[^ ^32^ ^]^ In our structures of Zn *Pf* Rd, these features are maintained at all temperatures (Figure 1g). In short, at higher temperatures, some H‐bonding networks change, while others are conserved.

Although the static X‐ray structures are similar, additional information is obtained by examination of the Cα mean square deviations reflected in the crystallographic B‐factors. Using blue for the least disorder and red for the regions with the largest deviations, the structures at 100 K and 393 K are compared in Figure 1h and Figure 1i.

### General Temperature‐Dependent Trends in Crystal Structures

**Figure 2:**
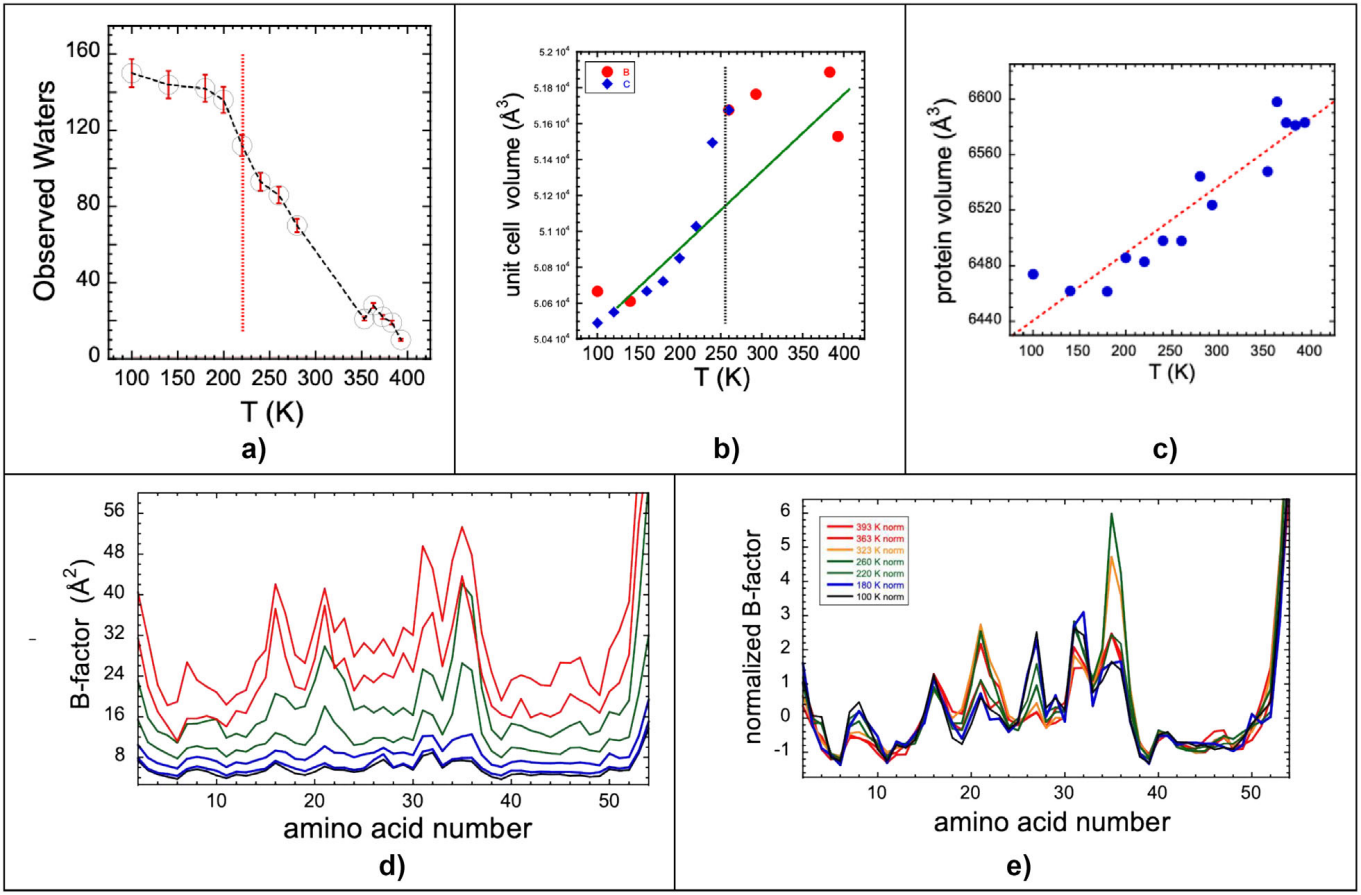
Crystal Properties. a) The number of crystallographically defined water molecules as a function of temperature. b) Unit cell volume changes versus temperature. c) Protein volume versus temperature. d) Plots of B‐factors vs. amino acid residue number for different temperatures. B‐factors rise monotonically with plotted temperatures of 100, 180, 220, 260, 323, 363, and 383 K. A chart showing B‐factors for all temperatures is included in Supporting Information. e) Same plot for “normalized” B‐factors.

Apart from focusing on individual structures, it is also useful to examine general trends in the Zn *Pf* Rd crystal properties. To illustrate how water mobility changes with temperature, in Figure 2a we plot the number of waters observed in diffraction structures as a function of temperature. The sharp drop‐off around 240 K is consistent with a protein dynamical transition involving “translational displacements of hydration water” as described by Doster.^[^ ^33^ ^]^ In Figure 2b, the unit cell volume is plotted as a function of temperature. From 100 to 393 K, we observed a 2.8% increase in the unit cell volume, somewhat less than the 3.9% expansion observed by Guerrero over 100 – 310 K for their STEP protein.^[^ ^34^ ^]^ There appears to be an inflection in the 230 K region; this may also reflect a dynamical transition in the crystal water properties. Finally, in Figure 2c, we display the protein volume versus temperature.^[^ ^35^ ^]^ These data translate to an expansion of 0.6%/100 K (0.48 per degree), similar to a value of 0.4%/100 K found by Tilton et al. for Ribonuclease‐A^[^ ^36^ ^]^

The B‐factors themselves are shown in Figure 2d and as “normalized” B‐factors in Figure 2e. At cryogenic temperatures (100 K and 140 K), the largest B‐factors (hence greatest disorder) occur near the N‐ and C‐ termini. In accord with previous work, the regions with the smallest B‐factors involve the “knuckles” that create the Zn‐binding site.^[^ ^37^ ^]^ The β‐sheets are also relatively immobile, as are the core aromatic sidechains. As the temperature increases to solution values, the highest amount of disorder is associated with Asp‐21 in loop A, as well as Asp‐34 and Asp‐35 in loop B. At high temperatures, 383 K and 393 K, there is large and comparable disorder across both loop regions.

### Temperature‐Dependent Molecular Dynamics (MD) of Zn *Pf* Rd

**Figure 3:**
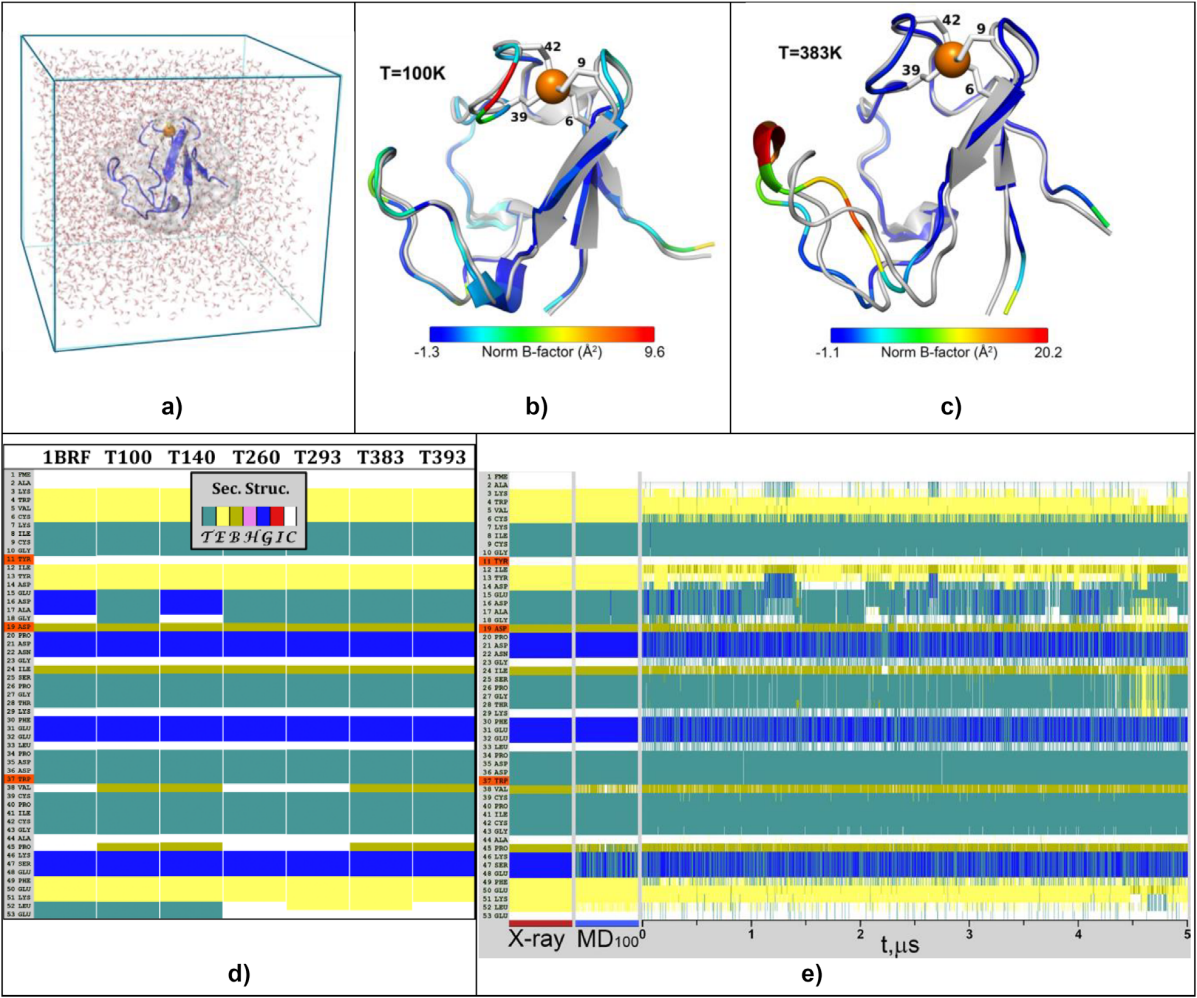
a) Setup for MD calculations. Box is 50 Å x 50 Å x 50 Å and contains 3300 water molecules. b) Overlay of 100 K XRD structure with median 100 K MD structure from 5 µs simulation. The MD structure is color coded according to RMSF fluctuations. c) Overlay of 393 K crystal structure with median 393 K MD structure from 5 µs simulation. d) Static secondary structure from STRIDE analysis.^[^ ^42^ ^]^ Single letter secondary structure codes: T: turn, E: extended sheet, B: isolated bridge, H: α‐helix, G: 3–10 helix, I: π‐helix, C: coil. e) Time‐dependent evolution of secondary structure over 5 µs MD along with 393 K x‐ray and 100 K MD inserts underscored by red and blue lines respectively.

*Pf* Rd has been studied by MD for nearly 30 years,^[^ ^31^, ^38^, ^39^ ^]^ beginning with 100 ps studies by Swartz and Ichiye in 1996 ^[^ ^38^ ^]^ and most recently studies of Rd unfolding in 2024.^[^ ^40^ ^]^ Over time, the duration of the calculations has improved, as have the quality of the protein force fields and the water models used in the simulations. As a complement to the x‐ray diffraction work, here we present MD on the 5 µs time scale. The setup, including cell size and water matrix, is shown in Figure 3a.

**Root‐Mean‐Square Fluctuations**. In Figure 3b,c we compare our x‐ray diffraction structures with 5 µs MD calculations at different temperatures, with the protein chains colored according to normalized B‐factors. The 100 K MD structure in Figure 3b is nearly superimposable with the x‐ray diffraction result. In Figure 3c, we see that the largest discrepancy between MD and XRD is near Asp‐21, at the turning point of loop A. In MD videos this loop can be seen to occasionally flex back and forth on the scale of several Å (SM1).

**Secondary Structure**. The secondary structure of *Pf* Rd has been described as comprised of “three 3_10_‐helices (residues 19–21, 29–31 and 45–47) and one antiparallel three‐stranded β‐sheet (residues 2–5, 11–13 and 48–50)”.^[^ ^41^ ^]^ We calculated the secondary structure from the STRIDE routine.^[^ ^42^ ^]^ In Figure 3d, we compare the static XRD secondary structure for temperatures from 100 K to 393 K. We see that the only stretch of multiple residues that changes is Glu‐15→Gly‐18, and this just around 100 K to 260 K. In Figure 3e we show the MD‐calculated time‐evolution of secondary structure for Zn *Pf* Rd at 100 K and 393 K. In contrast with the stability of the crystallographic assignments, now we see that the only stretches that do not fluctuate are around the metal binding knuckles (Lys‐7→Tyr‐11 and Cys‐39→Gly‐43) and a short stretch of Pro‐34→Trp‐37.

**Figure 4:**
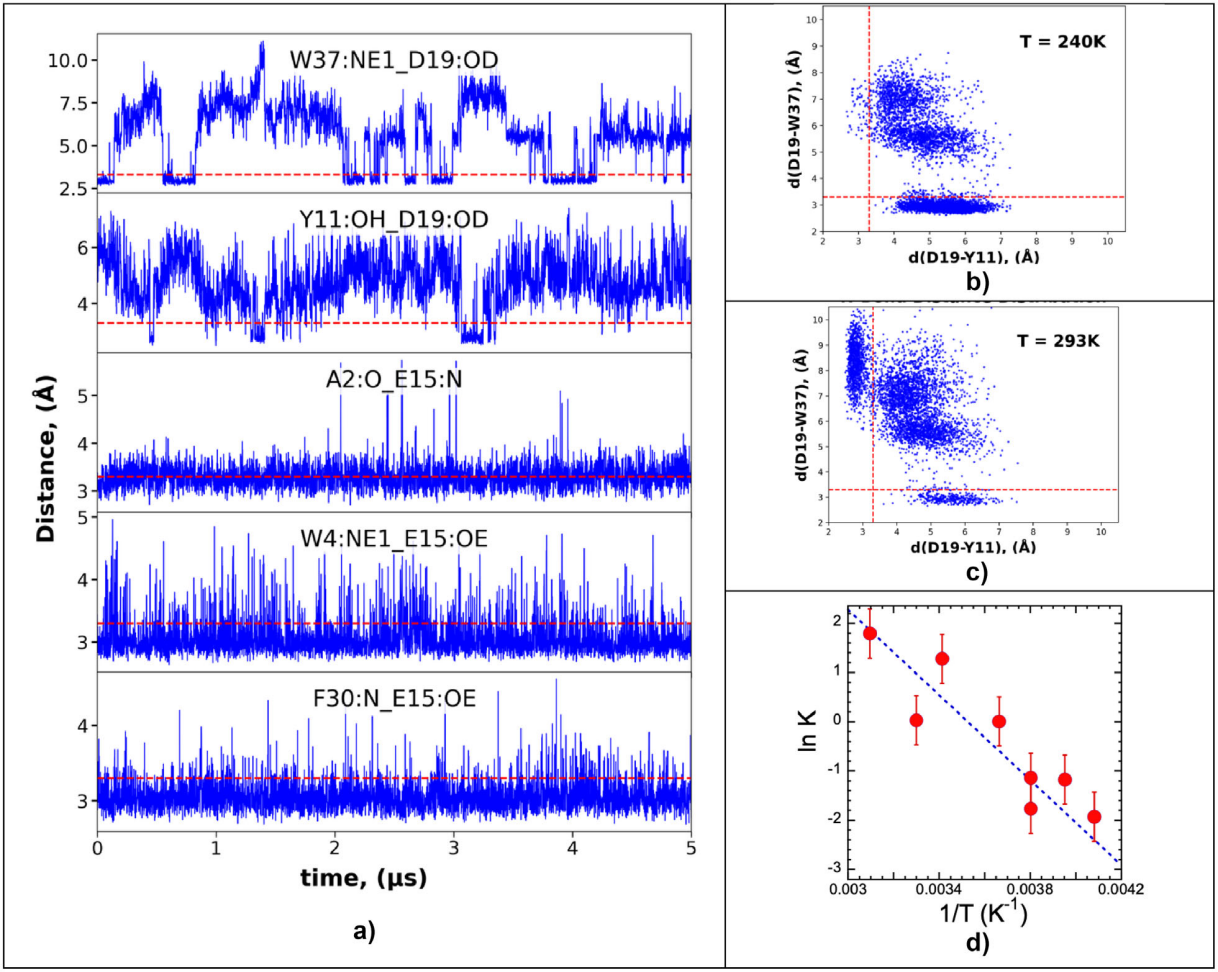
a) Time‐dependent distances at 253 K: (top to bottom) a. D19 O – W37 N^ε^; b. D19 O – Y11 OH; c. E15 N – A2 O; d. E15 O – W4:N^ε^; e. E15 O – F30 N. b) Distance scatter plot for 240 K D19 O^c^ – Y11 O versus D19 O^c^ – W37 N^ε^. c) Same distance scatter plot for 293 K. d) van't Hoff plot for D19‐Y11 versus D19‐W37 equilibrium. Error bars are based on reproducibility between different simulations.

The static pictures from x‐ray diffraction are complemented by examining the MD for both of the highlighted H‐bonding regions. In Figure 4a, for 253 K, we compare the distances of the Asp‐19 carboxylate O^c^ to two different H‐bonding partners: Trp‐37 N^ε^ and Tyr‐11 O, as well as distances around Glu‐15. Starting from the 353 K XRD structure, the MD shows that the H‐bonding pattern around Asp‐19 alternates between the two stable conformations suggested by XRD in Figure 2. The temperature‐dependent internal H‐bonding is summarized in Table S2.

Scatter plots are another way to show the MD results. At 240 K (Figure 4b), the dominant H‐bonded conformations (49%) retain a short Asp‐19‐O^c^ – Trp‐37‐N^ε^ distance. In the 293 K simulation (Figure 4c), the major H‐bonded conformation has a short Asp‐19‐O^c^ – Tyr‐11‐OH distance (29%), while a smaller subset retains a short Asp‐19‐O^c^ – Trp‐37‐N^ε^ distance (7%). In both cases a large fraction (∼50%–70%) of the calculated structures involves intermediates with both H‐bonds broken. Using the temperature‐dependent ratios of H‐bonded Tyr‐11 vs. Trp‐37, a van't Hoff plot was constructed to gauge the thermodynamics of the Asp‐19 H‐bonding equilibrium (Figure 4d). Over the range from 245 K to 323 K, this yielded *ΔH* = ∼31 kJ mol^−1^ and *ΔS* = 108 J K^−1^ mol^−1^; both with errors estimated as ± 10%. These are reasonable values for a modest conformational change.

## Discussion

X‐ray crystallography remains the premier technique for protein structure determination.^[^ ^43^ ^]^ This continues to be true despite enormous progress in alternative methods such as neutron diffraction, NMR, cryoelectron microscopy (cryo‐EM), and AI approaches such as AlphaFold and RoseTTAFold. The vast majority of the >200000 protein structures reported in the PDB are for x‐ray diffraction measurements under cryogenic conditions, generally ∼100 K, and cryo‐EM structures are often recorded in the ∼100 ± 20 K range. However, the possibility that low‐temperature structures are not representative of the situation under “real world” conditions is now well established.^[^ ^44^ ^]^

Progress in room temperature crystallography has revealed that there are often significant differences with measurements conducted at 100 K. For example, with the small protein crambin, six‐and seven‐membered rings of water molecules are observed at 100 K, but these disappear at 200 K, suggesting that can be considered “cryoartefacts”.^[^ ^45^ ^]^ Halle has noted that side‐chain conformations, hydration structures, ligand association, and proton dissociation equilibria may all be affected by flash‐cooling.^[^ ^45^ ^]^ In a similar vein, Fraser et al. found that “crystal cryocooling remodels the conformational distributions of more than 35% of side chains and eliminates packing defects necessary for functional motions”.^[^ ^46^ ^]^ Bradford and coworkers have argued that “RT structures can provide key insights that are not apparent in cryo structures”, since changing to room temperature (RT) can ″populate higher energy conformational states that are hidden under routine cryogenic conditions.^[^ ^44^ ^]^ With this growing realization of the limits of low‐temperature diffraction measurements, there has been noteworthy progress in room‐temperature crystallography^[^ ^34^, ^47^, ^48^, ^49^ ^]^ and the PDB now contains over 6000 structures around “room temperature” (>263 K).

By analogy with the divide between structures obtained under cryo or ambient conditions, one might ask if a similar rift occurs between room‐temperature and near‐boiling water conditions. In this vein, Jacobs et al. asked if “extremophilic proteins (should) be studied at these organisms’ temperatures >80 °C?”.^[^ ^48^ ^]^ Addressing this question requires experimental results, but there are only 10 PDB structures for temperatures at or above 323 K (50 °C) and the previous record was 90 °C.^[^ ^26^ ^]^ Access to our new high‐temperature structures for *Pf* Rd allows us to answer in the affirmative.

In our current work, we have found significant differences between the Zn *Pf* Rd structures obtained under cryo (100 K), ambient (260 K), and high‐T (393 K) conditions. Of special note is the H‐bonding for Asp‐19, which eventually switches from Trp‐37 to Tyr‐11 in the high‐T structure (Figure 1d–f). The MD calculations reveal that this switch is not static, but dynamic, with Asp‐19 swapping between both sites on the 100 ns timescale (Figure 4a). Despite the MD evidence for heterogeneity, the crystal structures invariably refined with a single conformation; perhaps crystal packing effects stabilize unique structures compared to solution MD which allows greater conformational freedom.

A proposal from Hernandez and LeMaster stands out as relevant to the current findings.^[^ ^50^ ^]^ They observed that the multiple turn region comprising residues 14–32 (our numbering) has conformational flexibility “comparable to that of the mesophile homologue, but by a means having a much lower temperature dependence thus allowing for net stability over a wider temperature range”.^[^ ^50^ ^]^ By x‐ray diffraction and molecular dynamics, we have also observed that loop A is a region with significant flexibility (Figure 3c). The opportunity for Asp‐19 H‐bonding to alternate between Trp‐37 and Tyr‐11 might serve to stabilize loop A despite its conformational flexibility.

From our comparisons of XRD data and MD calculations, it is clear that invoking a source of *Pf* Rd stability based on static crystal structures at a single temperature is fraught with dangers. Going forward, we plan additional comparisons with psychrophilic and mesophilic Rds under extreme conditions, by XRD, MD, and other methods. One can hope that this additional data will contribute to addressing this longstanding issue of *Pf* Rd stability.

### Summary and Conclusions

We have conducted X‐ray diffraction measurements and MD calculations on *Pyrococcus furiosus* rubredoxin from 100 K to 393 K. We observe changes for some features while others remain invariant. The H‐bonding from Asp‐19 to Trp‐37 is observed to switch to Tyr‐11 in x‐ray diffraction structures at high temperatures and this is confirmed in the MD calculations. The H‐bonding around Glu‐15 remains unperturbed in the crystal structures. Overall, these results show that discussions of extremophile protein stability and function under extreme conditions would benefit from structures obtained under those same conditions. Given that this is the first structure for an extremophile protein at above 100 °C, additional examples are needed to discern the magnitude and generality of structural changes under extreme conditions.

## Author Contributions

**S.P.C., F.J., I.L., T.D**.: Conceptualization. **I.L., T.D**.: Methodology. **S.P.C., F.J., I.L., T.D., D.G**.: Investigation. **I.L., T.D**.: Visualization. **S.P.C**.: Funding Acquisition. **S.P.C**.: Project Administration. **S.P.C., T.D**.: Supervision. **S.P.C**.: Writing — original Draft. **S.P.C., F.J., I.L., T.D**.: Writing — review & Editing.

## Conflict of Interests

The authors declare no conflict of interest.

## Supporting information



Supporting Information

Supporting Information

## Data Availability

The data that support this study are available from the corresponding authors upon request. The crystal structures for this study have been deposited in the Protein Data Bank.
