# Supplementary material for: Some Like It Hot –Structural Changes in Extremophile Rubredoxin at 120 °C
Source: Angew Chem Int Ed Engl. 2025 Nov 24;65(5):e20302. doi: 10.1002/anie.202520302 (PMC12851005; doi:10.1002/anie.202520302)
Supplement: Supplementary file 1 — Supporting Information [file ANIE-65-e20302-s002.pdf]

Supporting Information for

Some Like it Hot –
X-Ray Diffraction at 120 °C Reveals Structural Changes in Extremophile Protein

Tzanko Doukov [1], Igor Leontyev [2], Francis E. Jenney Jr.[3], Dominic George[2], Stephen P. Cramer[2]*
Corresponding author: spjcramer@seti.org

[1] SSRL, SLAC National Laboratory; Menlo Park, CA 94025 USA.
[2] SETI Institute; Mountain View, CA 94043 USA
[3] Georgia Campus, Philadelphia College of Osteopathic Medicine; Suwanee, GA 30024 USA

This PDF file includes:

Materials and Methods

Table S1. X-ray diffraction details.

Table S2. Intramolecular H-bonds at three temperatures.

SM Figure S1

SM Figure S2

SM References

Other Supplementary Materials for this manuscript include the following:

Movies

SM1. A QuickTime format movie of a 5 µs molecular dynamics simulation at 253 K showing the carboxylate sidechain of Zn *Pf* Rd Asp-19 oscillating between H-bonding with Tyr-11 and W-37. Simulation started with the 293 K x-ray structure.

Data

PDB files for 16 different Zn *Pf* Rd structures have been deposited in the Protein Data Bank as: 100K 9XZU (Ala N-terminus), 140K 9Y00, 180K 9Y0Y, 200K 9Y0Z, 220K 9Y11, 240K 9Y1U, 260K 9Y28, 280K 9Y29, 100K 9Y2E (fMet N-terminus), 293K 9Y2G, 313K 9Y38, 353K 9Y37, 363K 9Y33, 373K 9Y32, 383K 9Y30, and 393K 9Y2Y. Details in Table S1.

Materials and Methods

Production and purification of Zn Pf Rd.

The recombinant *Pyrococcus furiosus* rubredoxin (*Pf* Rd)was expressed in *E. coli* and purified essentially as described in (*1*). Recombinant *Pf* Rd is produced by *E. coli* with three N-terminal forms, N-fmet, N-met, N-ala (*1*). After a heat treatment to remove the majority of *E. coli* proteins from the cell extract and the initial purification by ion exchange and size exclusion chromatography, the *Pf* Rd is essentially pure, but still a mixture of the three N-terminal forms. The N-met form was separated from the other two using hydroxyapatite chromatography, and those two forms were finally separated by ion exchange chromatography on a higher resolution Source Q column (14). Zinc-substituted *Pf* Rd was prepared as described (*2*).

Crystallization of Zn Pf Rd

Zn-substituted *Pf* Rd with a concentration 40 mg/ml was crystallized by the hanging drop method with macro-seeding using the protocol originally used by Bau *et al.* (*3*).

X-Ray Diffraction

Crystal Mounting

We found that preparing the crystals at the beamline worked the best, since it did not demand cryogenic freezing procedure for storage. Rubredoxin crystals were covered with ParatoneN oil, and the surface water layer was scrapped away using Hampton nylon loop. Before mounting on the goniometer, excess oil was dabbed away leaving just the loop, the protein and an invisible lay of oil, thereby decreasing the excess background in the diffraction images.

Achieving high-temperature capabilities and temperature control

We utilized our previous experience with physiological range temperatures crystallography (*4*) (*5*). We used a cryo-annealer paddle at BL14-1 to isolate the crystal from continuous heating unrelated to the diffraction experiment. Multiple hardware updates allow us to use the Oxford Cryosystems Cryostream 1000 model N_2 cooler/heater (https://oxcryo.com/products/cryostream/) with a temperature range of 80–500 K at SSRL BL9-2 in a similar way. To avoid rapid protein crystal deterioration at high temperatures, we used the computerized shutdown of the heated N_2 gas flow up to 45 seconds, while the beamline hutch was searched and closed as well as the crystal is optically centered at the X-ray beam position. During these operations the crystal is at room temperature. Customized commands in the Blu-Ice control software (*6*) allowed multiple actions at the same time – keeping the annealing time counter, crystal centering, *etc.*

Control kinetic measurements showed that a J thermocouple at room temperature (~293 K) placed in a 373 K N_2 stream was within 0.5% of the desired temperature in ≤8 s and we used this equilibration time as a minimum criterion prior to data collection (see below). For data collection, when the software controlled annealer timer expired we started data collection within Blu-Ice, and data collection was initiated after a ~13 sec initiation check procedure. There is potential for improvement in the data collection protocol by decreasing the initiation time down to 8 seconds temperature equilibration delay (*5*).

Diffraction Data Collection

Zn-substituted *Pf* Rd crystals with dimensions 0.2 to 0.4 mm on each side were used for data collection. To achieve resolutions akin to those obtained at cryogenic temperatures, larger crystals are necessary for the acquisition of X-ray diffraction data at and above room temperature, as elevated temperatures might result in increased radiation damage (*7*) (*8*) (*9*) (*10*) (*11*) (*12*) (*13*) (*14*). To optimize diffraction intensity while reducing the quantity of absorbed photons per unit cell, the dimensions of the beam and crystal are aligned closely. We routinely used the largest beam size of 200 µm (horizontal) by 80 µm (vertical) at BL9-2 and 80 by 80 µm beam

at BL12-2. At least 100 degrees of rotation data were collected as quickly as possible with a rotation interval at least 1 degree per image.

Diffraction Data Processing

Diffraction data recorded on Pilatus 6M and Eiger 16M PAD detectors (*15*) was processed with the XDS (*16*) and Pointless (*17*) and Aimless (*18*), CCP4 (*19*), and STARANISO programs, as implemented in the autoPROC software (*20*).

Molecular Dynamics Calculations

MD simulations were done using GROMACS 2024.2 package (*21*).

Systems setup

All simulations of Zn-rubredoxin were setup using ZAFF parameters for zinc-cysteine co-factor and Amber19 force field for the rest of the protein as described in the ZAFF tutorial (*22*), using the tleap program of AmberTools23 (*23*). The protein structure for each MD studied temperature 100, 240, 293, 383 and 393 Kelvin was taken from the corresponding temperature X-ray structure obtained in the present work. MD runs for studying the T-dependence of D19 H-bonding shown in Figure 4 were started from the 293K X-ray structure for all temperatures except T=240K. The later run was started from the 240K X-ray structure. Protonation states of protein residues were determined by PropKa algorithm using the pdb2pqr server (*24*). The protein structure for each temperature was centered in a rectangular simulation box and solvated using TIP4P-Ew (*25*) water model leaving at least 10 Å distance between the protein and the box edges. The system was neutralized using Cl^- and Na^+ ions with the salt concentration 3.0 M which mimics the experimental Xray buffer salt concentration. The Amber format coordinate and topology files generated by tleap were then converted to GROMACS input files using the ParmEd python tool (*26*).

Systems equilibration

For all MD runs, an initial minimization was performed using the conjugated gradient algorithm and flexible variant of TIP4P-Ew model. The solvated protein systems then were equilibrated with the smaller timestep 0.5fs and protein heavy atoms positionally restrained (k = 2.5 kcal/mol/Å^2) in two steps. The first step was run in NVT ensemble and second step in NPT ensemble with Berendsen barostat at 1 bar and 0.5 ps coupling constant. Both equilibration steps were run for 1ns.

Production runs

All production simulations were run for 5 μs. A constant temperature at T=100K, 383 and 393Kwas maintained implicitly using the stochastic dynamics (sd) integrator and separate protein and solvent thermosttating. In T-dependence series (fig4) the leap-frog integrator was used and temperature was maintained by the **v-rescale** thermostat with stochastic term. The pressure was maintained at 1 bar using the Parrinello–Rahman barostat (*27*) with a coupling time constant of 5 ps. Bonds to all hydrogen atoms were constrained using LINKS (*28*) and the integration time step was set to 2 fs. Long-range electrostatic interactions were calculated using the Particle-Mesh Ewald method (*29*) with a real-space cutoff of 9 Å and grid spacing of 1.2 Å. Lennard-Jones interactions were cut off at 9 Å. The system coordinates were saved every 1 ns that resulted in 5000 frames in each trajectory.

**Protein**	**PfZnRd_fM1**	**PfZnRd_A2**	**PfZnRd_A2**	**PfZnRd_A2**	**PfZnRd_A2**	**PfZnRd_A2**	**PfZnRd_A2**	**PfZnRd_A2**
**PDB ID**	**9Y2E**	**9XZU**	**9Y00**	**9Y0Y**	**9Y0Z**	**9Y11**	**9Y1U**	**9Y28**
**Temperature [K]**	**100**	**100**	**140**	**180**	**200**	**220**	**240**	**260**
SSRL Beamline	BL9-2	BL12-2	BL12-2	BL12-2	BL12-2	BL12-2	BL12-2	BL12-2
Crystal Size (mm)	0.37,0.10, 0.10	0.30, 0.28, 0.25	0.30, 0.28, 0.25	0.30, 0.28, 0.25	0.30, 0.28, 0.25	0.30, 0.28, 0.25	0.30, 0.28, 0.25	0.30, 0.28, 0.25
Wavelength [Å]	0.88557	0.72929	0.72929	0.72929	0.72929	0.72929	0.72929	0.72929
Resolution range [Å]	34.40-0.78 (0.80-0.78)	26.93-0.82 (0.89-0.82)	26.95-0.83 (0.90-0.83)	26.97-0.85 (0.92-0.85)	26.98-0.86 (0.93-0.86)	27.03-0.91 (0.99-0.91)	27.11-0.98 (1.05-0.98)	27.17-1.01 (1.09-1.01)
Space group	$P2_12_12_1$	$P2_12_12_1$	$P2_12_12_1$	$P2_12_12_1$	$P2_12_12_1$	$P2_12_12_1$	$P2_12_12_1$	$P2_12_12_1$
Unit cell : a, b, c (Å)	33.96 34.40 43.37	33.86 34.33 43.43	33.89 34.35 43.47	33.92 34.36 43.52	33.96 34.38 43.56	33.97 34.44 43.62	34.03 34.58 43.68	34.09 34.70 43.69
Total reflections	142941 (2429)	250821 (10164)	244108 (10485)	233020 (11003)	225327 (11233)	187355 (9548)	153808 (7420)	139569 (6287)
Unique reflections	55180 (1753)	38521 (1926)	37292 (1865)	35436 (1772)	34125 (1706)	28318 (1416)	23294 (1165)	21139 (1057)
Multiplicity	2.6 (1.6)	6.5 (5.3)	6.5 (5.6)	6.6 (6.2)	6.6 (6.6)	6.6 (6.7)	6.6 (6.4)	6.6 (5.9)
Completeness (spherical) (%)	95.7 (61.0)	77.9 (19.1)	77.2 (18.4)	78.3 (20.3)	77.9 (19.7)	76.9 (19.3)	76.9 (20.9)	76.0 (19.4)
Completeness (ellipsoidal) (%)		93.2 (51.3)	93.6 (53.6)	94.0 (58.8)	94.1 (60.5)	94.0 (60.4)	93.6 (58.6)	93.6 (56.4)
Mean I/sigma(I)	10.4 (0.5)	17.5 (1.6)	17.4 (1.6)	17.2 (1.6)	16.8 (1.6)	16.1 (1.4)	15.9 (1.4)	15.8 (1.4)
Wilson B-factor [$Å^2$]	8.8	9.23	9.66	10.05	10.56	11.34	12.31	12.64
R-merge	0.036 (1.34)	0.049 (0.68)	0.048 (0.716)	0.047 (0.745)	0.048 (0.793)	0.049 (0.931)	0.048 (0.886)	0.049 (0.948)
R-pim	0.033 (1.34)	0.020 (0.32)	0.020 (0.326)	0.020 (0.320)	0.020 (0.332)	0.020 (0.381)	0.020 (0.374)	0.021 (0.412)
$CC_{1/2}$	0.998 (0.114)	0.999 (0.753)	1.000 (0.75)	1.000 (0.782)	0.999 (0.755)	1.000 (0.728)	0.999 (0.728)	0.999 (0.658)
ISa	26.65	45.07	44.4	45.02	41.91	40.92	41.2	39.9
Mosaicity	0.14	0.14	0.14	0.15	0.15	0.17	0.18	0.18
**Refinement**								
Refinement range	26.95-0.78 (0.80-0.78)	26.93-0.82 (0.85-0.82)	26.95-0.83 (0.85-0.83)	26.97-0.85 (0.87-0.85)	26.98-0.86 (0.88-0.86)	27.03-0.91 (0.94-0.91)	27.11-0.98 (1.00-0.98)	27.17-1.01 (1.04-1.01)
R_{work} (%)	13.2 (43.5)	13.2 (33.8)	12.9(34.5)	13.1 (36.3)	13.6 (39.0)	12.7 (32.1)	12.6 (29.7)	12.2 (30.9)
R_{free} (%)	13.9 (43.5)	14.8 (46.0)	13.9 (32.2)	15.0 (50.2)	14.9 (42.3)	14.7 (32.8)	14.8 (41.5)	14.3 (63.4)
**No. of non-H atoms**								
Total	597	602	592	583	584	570	544	520
Macromolecules	439	432	432	426	435	450	445	433
Ligands (Zn, K)	1	1, 1	1,1	1,1	1,1	1,1	1,1	1

Water	157	168	158	153	147	118	97	86
**R.m.s. deviations**								
Bond Lengths [Å]	0.013	0.013	0.013	0.013	0.012	0.013	0.012	0.012
Angles [°)]	1.816	1.887	1.835	1.895	1.926	2.028	2.123	2.02
Average B Factor [Å^2]	11.535	10.118	12.328	10.824	12.569	13.42	16.01	17.98
Macromolecules	8.32	7.07	8.288	7.924	8.647	10.59	13.44	15.29
Ligands (Zn, K)	4.27, 13.50	3.94, 11.18	4.49, 12.05	4.70, 9.25	5.03, 10.98	6.33, 19.59	7.73, 27.57	8.61
Water	20.535	17.99	23.424	19.005	24.225	24.258	27.874	31.54
Clashscore	0	0	0	3.67	0	2.25	2.3	1.19
MolProbity score	0.5	0.5	0.5	1.16	0	1	1.01	0.83
**Ramachandran Plot**								
Favored (%)	100	100	100	98.04	98.04	100	100	100
Allowed (%)	0	0	0	1.96	1.96	0	0	0
Outliers (%)	0	0	0	0	0	0	0	0

**Protein**	**PfZnRd_A2**	**PfZnRd_fM1**	**PfZnRd_fM1**	**PfZnRd_fM1**	**PfZnRd_fM1**	**PfZnRd_fM1**	**PfZnRd_fM1**	**PfZnRd_fM1**
**PDB ID**	**9Y29**	**9Y2G**	**9Y38**	**9Y37**	**9Y33**	**9Y32**	**9Y30**	**9Y2Y**
**Temperature [K]**	**280**	**293**	**313**	**353**	**363**	**373**	**383**	**393**
SSRL Beamline	BL12-2	BL9-2	BL9-2	BL9-2	BL9-2	BL9-2	BL9-2	BL9-2
Crystal Size (mm)	0.30, 0.28, 0.25	0.40, 0.20, 0.19	0.45, 0.24, 0.20	0.25, 0.15, 0.15	0.50, 0.25, 0.25	0.32, 0.25, 0.25	0.25 ,0.25, 0.20	0.38, 0.23, 0.20
Wavelength [Å]	0.72929	0.97946	0.97946	0.97946	0.97946	0.97946	0.97946	0.97946
Resolution range [Å]	27.30-1.08 (1.19-1.08)	27.17-1.14 (1.26-1.14)	27.33-1.41 (1.52-1.41)	26.81-1.84 (1.87-1.84)	27.08-1.35 (1.48-1.35)	26.97-1.47 (1.60-1.47)	27.11-1.53 (1.64-1.53)	27.06–1.68 (1.96 -1.68)
Space group	$P2_12_12_1$	$P2_12_12_1$	$P2_12_12_1$	$P2_12_12_1$	$P2_12_12_1$	$P2_12_12_1$	$P2_12_12_1$	$P2_12_12_1$
Unit cell : a, b, c (Å)	34.22 34.93 43.78	34.13 34.69 43.73	34.36 34.92 43.92	34.02 34.49 42.62	34.22 34.70 43.31	34.16 34.63 42.98	34.27 34.81 43.21	34.24 34.76 43.11
Total reflections	109790 (5028)	162291 (6625)	107698 (5704)	19828 (962)	114085 (5178)	81430 (4560)	85352 (5005)	38826 (2002)
Unique reflections	16617 (831)	14424 (721)	8608 (430)	4637 (230)	9160 (458)	7348 (367)	6867 (343)	3582 (199)
Multiplicity	6.6 (6.1)	11.3 (9.2)	12.5 (13.3)	4.3 (4.2)	12.5 (11.3)	11.1 (12.4)	12.4 (14.6)	10.8 (10.1)
Completeness (spherical) (%)	72.8 (15.5)	73.9 (14.2)	81.3 (21.1)	98.9 (100.0)	76.9 (16.0)	80.9 (18.9)	83.5 (22.9)	57.8 (8.9)
Completeness (ellipsoidal) (%)	91.0 (51.9)	91.3 (48.2)	90.7 (42.4)		91.5 (46.0)	90.7 (37.1)	90.4 (38.8)	87.2 (47.8)
Mean I/sigma(I)	13.2 (1.6)	9.1 (1.6)	11.0 (1.3)	4.0 (0.8)	13.3 (1.5)	12.0 (1.5)	7.7 (1.5)	4.8 (1.9)
Wilson B-factor [Å^2]	15.17	21.67	29.17	30.76	27.7	29.74	30.77	29.34

R-merge	0.056 (0.938)	0.134 (2.238)	0.111 (1.635)	0.223 (1.652)	0.098 (2.046)	0.105 (2.145)	0.337 (4.954)	0.421 (1.936)
R-pim	0.024 (0.408)	0.042 (0.750)	0.033 (0.460)	0.118 (0.856)	0.031 (0.629)	0.033 (0.631)	0.104 (1.345)	0.135 (0.630)
$CC_{1/2}$	0.999 (0.671)	0.993 (0.502)	0.997 (0.685)	0.984 (0.419)	0.994 (0.426)	0.999 (0.567)	0.965 (0.170)	0.977 (0.636)
ISa	36.8	11.26	14.79	8.59	17.01	21.42	3.55	6.63
Mosaicity	0.2	0.32	0.41	0.33	0.38	0.55	0.26	0.23
**Refinement**								
Refinement range	27.30-1.08 (1.11-1.08)	27.17-1.14 (1.26-1.14)	27.33-1.41 (1.45-1.41)	26.83-1.84 (1.88-1.84)	27.08-1.35 (1.38-1.35)	26.97-1.47 (1.51-1.47)	27.17-1.61 (1.65-1.61)	27.06-1.68 (1.73-1.68)
R_{work} (%)	12.6 (38.8)	13.8 (34.6)	15.3 (35.6)	19.1 (34.3)	11.8 (52.5)	12.6 (43.9)	15.5 (32.1)	21.5 (41.0)
R_{free} (%)	14.4 (30.6)	15.8 (7.6)	19.5 (0.0)	25.3 (30.5)	16.0 (0.0)	17.3 (52.1)	17.3 (28.9)	24.3 (41.2)
**No. of non-H atoms**								
Total	514	476	471	466	470	467	443	423
Macromolecules	442	423	423	443	443	444	423	422
Ligands (Zn, K)	1	1	1	1	1, 1	1	1	1
Water	71	52	47	22	25	22	19	10
**R.m.s. deviations**								
Bond Lengths [Å]	0.011	0.011	0.009	0.006	0.011	0.009	0.008	0.009
Angles [°)]	1.904	1.845	1.701	1.815	1.795	1.664	1.692	1.801
Average B Factor [Å^2]	19.02	22.44	28.618	32.305	32.012	30.526	30.812	35.151
Macromolecules	16.75	20.52	27.042	32.235	31.214	30.052	30.384	35.209
Ligands (Zn, K)	8.99	10.65	15.57	18.88	16.79, 39.43	16.8	16.4	20.1
Water	33.16	38.03	42.835	33.72	46.226	39.687	40.352	32.724
Clashscore	2.3	0	0	0	2.3	1.15	0	2.48
MolProbity score	1.01	0	0.5	0.74	1.24	1.06	0.5	1.79
**Ramachandran Plot**								
Favored (%)	**PfZnRd_A3**	**PfZnRd_fM1**	**PfZnRd_fM1**	**PfZnRd_fM1**	**PfZnRd_fM1**	**PfZnRd_fM1**	**PfZnRd_fM1**	**PfZnRd_fM1**
Allowed (%)	**9Y30**	**9Y2G**	**9Y38**	**9Y37**	**9Y33**	**9Y32**	**9Y30**	**9Y2Y**
Outliers (%)	0	0	0	0	0	0	0	0

Table S1. Data collection, processing, and refinement statistics for Zn *Pf* Rd at various temperatures.

	100 K	323 K	393 K
	Trp4-$N^{\varepsilon1}$ – Glu15-$O^{\varepsilon2}$	Trp4-$N^{\varepsilon1}$ – Glu15-$O^{\varepsilon2}$	Lys3-N^{ζ} – Asp14-$O^{\delta2}$
	Lys7-N^{ζ} –Glu50-O $^{\varepsilon1}$	Lys7-N^{ζ} –Glu50-$O^{\varepsilon1}$	Trp4-$N^{\varepsilon1}$ – Glu15-$O^{\varepsilon2}$
	Asn22-$N^{\delta2}$ – Asp19-$O^{\delta1}$	Asn22-$N^{\delta2}$ – Asp19-$O^{\delta2}$	Asn22-$N^{\delta2}$ – Asp19-$O^{\delta2}$
	Trp37- $N^{\varepsilon1}$ – Asp19-$O^{\delta2}$	Trp37- $N^{\varepsilon1}$ – Asp19-$O^{\delta2}$	Tyr11-OH – Asp19--$O^{\delta2}$
	Ser47-O^{γ} – Glu48-$O^{\varepsilon2}$	Ser47-O^{γ} – Glu48-$O^{\varepsilon2}$	

Table S2. Intramolecular H-bonds at three temperatures

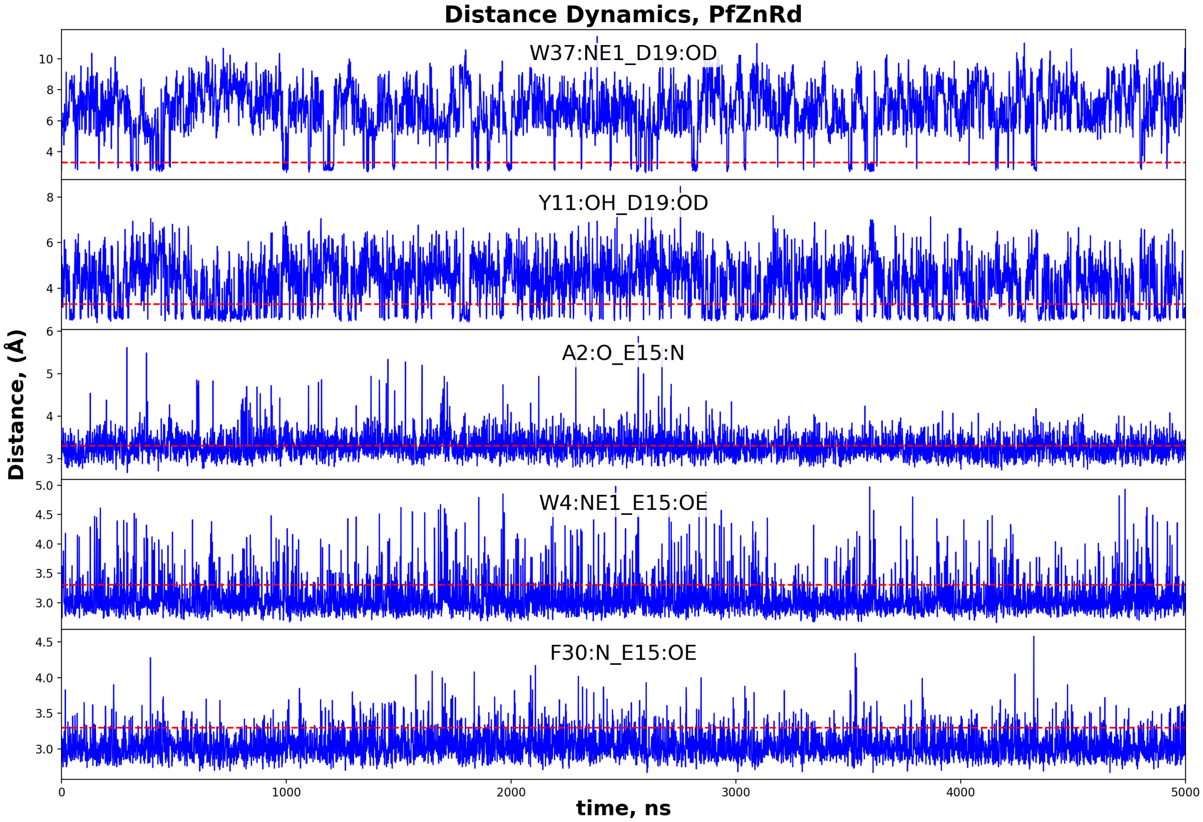

Figure S1. Dynamics at 293 K.

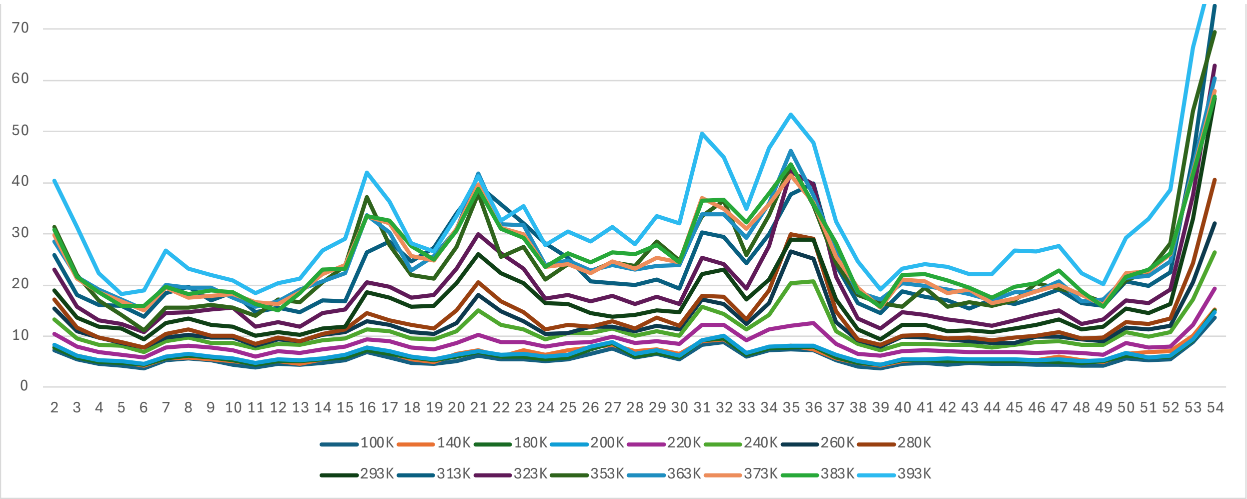

Figure S2. A chart of B-factors *vs*. amino acid position for 16 different temperatures.

References

1. F. E. Jenney, Jr., M. W. W. Adams, Rubredoxin from *Pyrococcus furiosus*. *Meth. Enzymol.,* **334**, 45-55 (2001).
2. E. R. Zartler *et al.*, Structural Basis for Thermostability in Aporubredoxins from *Pyrococcus furiosus* and *Clostridium pasteurianum*. *Biochemistry,* **40**, 7279-7290 (2001).
3. R. Bau *et al.*, Crystal structure of rubredoxin from *Pyrococcus furiosus* at 0.95 Å resolution, and the structures of N-terminal methionine and formylmethionine variants of *Pf* Rd. Contributions of N-terminal interactions to thermostability. *J. Biol. Inorg. Chem.,* **3**, 484-493 (1998).
4. S. Du *et al.*, Chapter Eight - Refinement of multiconformer ensemble models from multi-temperature X-ray diffraction data. *Meth. Enz.,* **688**, 223-254 (2023).
5. T. Doukov, D. Herschlag, F. Yabukarski, Instrumentation and experimental procedures for robust collection of X-ray diffraction data from protein crystals across physiological temperatures. *J. App. Cryst.,* **53**, 1493-1501 (2020).
6. T. M. McPhillips *et al.*, Blu-Ice and the Distributed Control System: software for data acquisition and instrument control at macromolecular crystallography beamlines. *J. Syn. Rad.,* **9**, 401-406 (2002).
7. C. Nave, E. F. Garman, Towards an understanding of radiation damage in cryocooled macromolecular crystals. *J. Syn. Rad.,* **12**, 257-260 (2005).
8. E. F. Garman, R. L. Owen, Cryocooling and radiation damage in macromolecular crystallography. *Acta Cryst. D,* **62**, 32-47 (2006).
9. R. J. Southworth-Davies, M. A. Medina, I. Carmichael, E. F. Garman, Observation of Decreased Radiation Damage at Higher Dose Rates in Room Temperature Protein Crystallography. *Structure,* **15**, 1531-1541 (2007).
10. M. Warkentin, R. E. Thorne, Glass transition in thaumatin crystals revealed through temperature-dependent radiation-sensitivity measurements. *Acta Cryst. D,* **66**, 1092-1100 (2010).
11. C. Rajendran, F. S. N. Dworkowski, M. Wang, C. Schulze-Briese, Radiation damage in room-temperature data acquisition with the PILATUS 6M pixel detector. *J. Sync. Rad.,* **18**, 318-328 (2011).
12. M. Warkentin, R. Badeau, J. Hopkins, R. E. Thorne, Dark progression reveals slow timescales for radiation damage between T = 180 and 240 K. *Acta Cryst. D,* **67**, 792-803 (2011).
13. P. Roedig *et al.*, Room-temperature macromolecular crystallography using a micro-patterned silicon chip with minimal background scattering. *J. App. Cryst.,* **49**, 968-975 (2016).
14. E. F. Garman, M. Weik, in *Protein Crystallography: Methods and Protocols,* A. Wlodawer, Z. Dauter, M. Jaskolski, Eds. (Springer New York, New York, NY, 2017), pp. 467-489.
15. A. Casanas *et al.*, EIGER detector: application in macromolecular crystallography. *Acta Cryst. D,* **72**, 1036-1048 (2016).
16. W. Kabsch, XDS. *Acta Cryst. D,* **66**, 125-132 (2010).
17. P. Evans, Scaling and assessment of data quality. *Acta Cryst. D,* **62**, 72-82 (2006).
18. P. R. Evans, G. N. Murshudov, How good are my data and what is the resolution? *Acta Cryst. D,* **69**, 1204-1214 (2013).
19. J. Agirre *et al.*, The CCP4 suite: integrative software for macromolecular crystallography. *Acta Cryst. D,* **79**, 449-461 (2023).
20. C. Vonrhein *et al.*, Data processing and analysis with the autoPROC toolbox. *Acta Cryst. D,* **67**, 293-302 (2011).
21. M. J. Abraham *et al.*, GROMACS: High performance molecular simulations through multi-level parallelism from laptops to supercomputers. *SoftwareX,* **1-2**, 19-25 (2015).
22. P. Li, K. M. Merz Jr. (2015).

23. D. A. Case *et al.*, AmberTools. *J. Chem. Info. Model.,* **63**, 6183-6191 (2023).
24. E. Jurrus *et al.*, Improvements to the APBS biomolecular solvation software suite. *Prot. Sci.,* **27**, 112-128 (2018).
25. H. W. Horn *et al.*, Development of an improved four-site water model for biomolecular simulations: TIP4P-Ew. *J. Chem. Phys.,* **120**, 9665-9678 (2004).
26. M. R. Shirts *et al.*, Lessons learned from comparing molecular dynamics engines on the SAMPL5 dataset. *J. Comp.-Aided Mol. Des.,* **31**, 147-161 (2017).
27. M. Parrinello, A. Rahman, Polymorphic transitions in single crystals: A new molecular dynamics method. *J. App. Phys.,* **52**, 7182-7190 (1981).
28. B. Hess, P-LINCS: A Parallel Linear Constraint Solver for Molecular Simulation. *J. Chem. Theory Comp.,* **4**, 116-122 (2008).
29. T. Darden, D. York, L. Pedersen, Particle mesh Ewald: An N·log(N) method for Ewald sums in large systems. *J. Chem. Phys.,* **98**, 10089-10092 (1993).
